# The Correlation between Altmetric Attention Score and Traditional Bibliometrics in Top Nursing Journal Articles

**DOI:** 10.1155/2023/2789960

**Published:** 2023-02-22

**Authors:** Lingmin Chen, Mutong Yang, Nian Li, Ying He, Yonggang Zhang

**Affiliations:** ^1^Department of Anesthesiology and National Clinical Research Center for Geriatrics, West China Hospital, Sichuan University, Chengdu 610041, China; ^2^West China Hospital of Stomatology, Sichuan University, Chengdu 610041, China; ^3^Department of Medical Administration, West China Hospital, Sichuan University, Chengdu 610041, China; ^4^Department of Integrated Traditional and Western Medicine, West China Hospital, Sichuan University, Chengdu 610041, China; ^5^Department of Periodical Press/National Clinical Research Center for Geriatrics, West China Hospital, Sichuan University, Chengdu 610041, China; ^6^Chinese Evidence-Based Medicine Center, West China Hospital, Sichuan University, Chengdu 610041, China; ^7^Nursing Key Laboratory of Sichuan Province, Chengdu 610041, China

## Abstract

**Background:**

Altmetric Attention Score (AAS) is a quantitative measurement of the online impact of research and has a potential correlation with traditional bibliometrics. However, the correlation for nursing journal articles is still unknown. The objective of the study was to analyze the correlation between AAS and traditional bibliometrics in the top nursing journal articles.

**Materials and Methods:**

Articles published in top nursing journals (the journals with the top 20 5-year impact factors) from 2010 to 2019 were included. The correlations between AAS and citations, AAS and Relative Citation Ratio (RCR) score, AAS and Category Normalized Citation Impact (CNCI) value, and AAS and impact factors were analyzed. Statistical analyses were performed using Stata 25.0 software.

**Results:**

A total of 15,212 journal articles were included in the study. Very weak correlations were found between AASs and citations [0.124 (95% CI, 0.108–0.14)], AASs and RCRs [0.26 (95% CI, 0.244–0.275)], and AASs and CNCIs [0.207 (95% CI, 0.192–0.223)]. The weak correlations were also found between AASs and impact factors in several journals. The weak correlations between AASs and citations, AASs and CNCIs, and AASs and RCRs were also found for most journals based on subgroup analysis.

**Conclusions:**

There is very weak correlations between AASs and traditional bibliometrics in top nursing journal articles. More studies should be conducted to assess how AAS influence bibliometrics, and how they can help manage nursing journal articles and research.

## 1. Introduction

Bibliometrics is the use of statistical methods to analyze publications, especially for scientific contents [1, 2]. It is used to justify the role of the researchers and the research team [3] and also to identify key partners [4] and the progress of a specific research field [5]. The most widely used bibliometrics method is based on citations [6], such as citation times, impact factors [7], *H* index [8], Eigenfactor score [9], and CiteScore [9]. However, these metrics have limitations. It would take time to accumulate citation times [10], and different journal articles could not be compared directly even in the same research field [11, 12]. Absolute citation times favor older journal articles and have limited utility in comparing journal articles from different fields [12, 13]. Thus, new metrics have been developed. RCR [14], developed by NIH, is defined as the total number of citations that a paper received per year divided by the average field-specific citation rate for a peer companion group [14, 15]. It allows the comparison of articles in different fields. CNCI is another metrics to assess the paper's impact and has been analyzed in different studies [16, 17], which can also be used to assess scientific articles from different research fields [16, 18].

In recent years, with the development of digital technology and the use of online platforms to discuss research, alternative-level metrics (altmetrics) have been introduced in research fields [12–14]. They were used to measure the journal articles with attention, dissemination, overall influence, and impacts [14]. The main altmetrics platform is Altmetric [19, 20], which compiles the number of mentions of a paper across the most used social media platforms such as Twitter, Facebook, and LinkedIn, and public policy documents, mainstream media, online reference managers, and other online platforms, to generate a weighted score, Altmetric Attention Score (AAS) [14]. AAS is a dynamic bibliometric which captures the online impact of a paper. It has been used to assess the online impact of journal articles across different fields [7, 14, 21] and provide potential evidence for research impact or journal strategy.

Nursing is an important discipline, and research helps the progress of the discipline by improving nursing practice and finally helping nursing management. Factors that influence the characteristics of nursing journal articles could further help journal articles to be quickly transformed into nursing practice and applied to nursing management. It has been reported that bibliometric analysis might help nursing management and nursing practice, and thus some studies have been performed in the field of nursing management [2, 16]. However, these studies did not analyze the important impact of online platforms on nursing management. It is still unclear whether the online impact would be associated with traditional bibliometrics and whether the online impact would help nursing paper citations. Furthermore, it is also unclear whether the nursing manager could use social media, public research platforms, or online platfroms to help nursing research impact. Although several articles have used bibliometrics methods to assess the top cited [2, 16] or the top impact nursing articles [2, 16], whether impact from social media, public research platforms, or online platforms will help higher citations is a mystery. Therefore, in the current study, we analyzed the Altmetric and bibliometric data of journal articles published in nursing journals, to assess the correlation between AASs and the citation metrics.

## 2. Methods

### 2.1. Inclusion and Exclusion Criteria for the Selection of Nursing Journal Articles

The inclusion criteria were as follows: (1) the journal articles should be published in top nursing journals. Five-year impact factors were used to identify the top nursing journals, and thus the top 20 5-year impact factor journals in the nursing category in the JCR report in 2021 were used in the current study. (2) The journals should be indexed by PubMed. Thus, the journals included in the study were as follows: Australian Critical Care, Birth-Issues in Perinatal Care, BMC Nursing, European Journal of Cardiovascular Nursing, Intensive And Critical Care Nursing, International Journal of Mental Health Nursing, International Journal of Nursing Studies, International Nursing Review, Journal of Advanced Nursing, Journal of Clinical Nursing, Journal of Nursing Management, Journal of Nursing Scholarship, Nurse Education Today, Nursing Ethics, Nursing Outlook, Research in Nursing & Health, Seminars in Oncology Nursing, Women and Birth, Worldviews on Evidence-Based Nursing, and Asian Nursing Research. (3) The paper type should be an article or review. (4) The paper could be found in Incites, iCite, Altmetric, and PubMed. The following exclusion criteria were used: (1) duplicated journal articles; (2) mismatched journal articles; (3) editorial articles, corrections, and letters.

### 2.2. Paper Searching

The journal articles were searched by the selected journal names in the three databases (Incites [16], iCite [14], and Altmetric [12]). All search results were downloaded. If any data for an article was missing, the article was further searched in PubMed or the specific journal websites.

### 2.3. Paper Screening and Data Extraction

All articles were screened according to the inclusion and exclusion criteria. Only articles which had all the required data (RCR, citation time, AAS, and CNCI) were included. The following information was extracted from each article: journal, author, impact factor, RCR score, citation time, CNCI value, AAS, etc. The RCR scores were extracted from the downloaded file from the iCite database, the CNCI values were extracted from the downloaded file from the Incites database, and the AASs were extracted from the downloaded file from the Altmetric database. The journals' impact facotrs were downloaded from the Web of Science.

### 2.4. Statistical Analysis

The characteristics of the included studies were analyzed. The correlations between AASs and citations, AASs and RCRs, AASs and CNCIs, and AASs and impact factors, were analyzed using Spearman correlation coefficients [12]. Subgroup analyses were performed according to journals, and years. All analyses were performed by SPSS 25.0 software.

## 3. Results

### 3.1. The Main Characteristics of Included Journal Articles

The data were collected by September 9^th^, 2022. After screening all data of the journal articles according to the inclusion and exclusion criteria, a total of 15,212 journal articles were included. The impact factors for all the selected 20 journals from 2010 to 2019 are shown in Table 1, and the impact factors of most journals increased from 2010 to 2019. The average citation of all journal articles was 17.36 times, and the median citation was 11 times. A total of 318 journal articles were cited 0 times, 14,059 journal articles were cited 1 to 50 times, 660 were cited 51 to 100 times, and 175 journal articles were cited more than 100 times.

### 3.2. The Performance of Included Journal Articles

The average RCR score of included articles was 1.71, and the median RCR score was 1.14. The RCR scores of 258 articles were 0; 6,576 articles had RCR scores between 0 and 1; 8,236 journal articles had RCR scores between 1 and 10; the RCR scores of 142 journal articles were higher than 10. The average CNCI value was 1.52, and the median CNCI value was 1.05. The CNCI values of 318 journal articles were 0; 7,017 journal articles had CNCI values between 0 and 1; 7,785 journal articles had CNCI values between 1 and 10; the CNCI values of 92 journal articles were higher than 10. The average AAS was 8.92, and the median AAS was 3. The AASs of 2264 journal articles were 0; 9,950 journal articles had AASs between 0 and 10; 2,866 journal articles had AASs between 10 and 100; the AASs of 132 journal articles were higher than 100. The citations, RCRs, CNCIs, and AASs of all journal articles in the 20 journals were summarized in listed in Table 2.

### 3.3. The Contribution of Different Sources to AAS

The sources that contributing to the AAS were individually analyzed from 2010 to 2019. For all years, Twitter was the most frequent contributor to AAS followed by news outlets and Policy (Table 3).

### 3.4. The Correlation of AASs with Citations, RCRs, CNCIs

**Figure 1:**
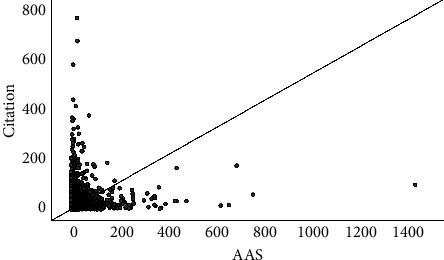
The correlation between AAS and citations.

**Figure 2:**
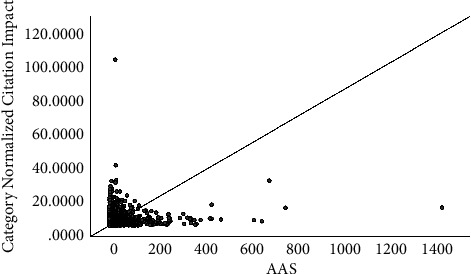
The correlation between AAS and CNCIs.

**Figure 3:**
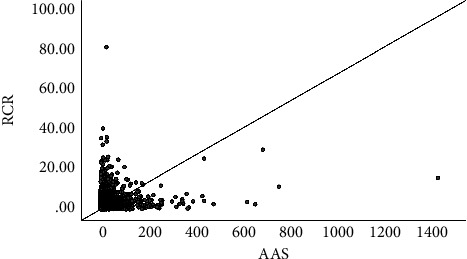
The correlation between AAS and RCRs.

The correlations of AASs with citations, CNCIs, and RCRs are shown in Figures 1–3. In summary, the correlation coefficient was 0.124 (95% CI, 0.108–0.14) between AASs and citations, 0.26 (95% CI, 0.244–0.275) between AASs and RCRs, and 0.207 (95% CI, 0.192–0.223) between AASs and CNCIs. The correlations for each journal are shown in Table 4.

For the International Journal of Nursing Studies, which ranked first in the JCR report (2021) with an impact factor of 6.612, a total of 1,437 journal articles were included. The total citation times was 43,729 (ranging from 0 to 582 by paper, median 19), the total AAS was 12,975 (ranging from 0 to 703 by paper, median 3), and the total RCR score was 3,985.92 (ranging from 0 to 35.94 by paper, median 1.79). Very weak correlations were found: 0.207 (95% CI, 0.158–0.258) between AASs and citations, 0.371 (95% CI, 0.325–0.418) between AASs and RCRs, and 0.267 (95% CI, 0.219–0.317) between AASs and CNCIs.

The Journal of Clinical Nursing ranked fourth in the JCR report (2021) with an impact factor of 4.423, from which we collected the highest number of journal articles in the current study. The total number of citations was 48,236 (ranging from 0 to 262 by paper, median 10), the total AASs was 17,842 (ranging from 0 to 251 by paper, median 2), and the total RCR score was 4,709.72 (ranging from 0 to 21.32 by paper, median 0.98). Very weak correlations were found: 0.133 (95% CI, 0.097–0.166) between AASs and citations, 0.312 (95% CI, 0.28–0.346) between AASs and RCRs, and 0.23 (95% CI, 0.199–0.264) between AASs and CNCIs.

The Journal of Nursing Management was the only management journal in the field of nursing, from which a total of 783 journal articles were included. The total number of citations was 13,906 (ranging from 0 to 203 by paper, median 11), the total AASs was 3,007 (ranging from 0 to 228 by paper, median 1), and the total RCR score was 1,593.84 (ranging from 0 to 17.58 by paper, median 1.43). Very weak correlations were found: 0.158 (95% CI, 0.086–0.229) between AASs and citations, 0.239 (95% CI, 0.162–0.304) between AASs and RCRs, and 0.206 (95% CI, 0.137–0.274) between AASs and CNCIs.

Nurse Education Today was the only education journal in the field of nursing, from which a total of 1,566 journal articles were analyzed. The total citation was 26,555 (ranging from 0 to 770 by paper, median 11), the total AAS was 15,837 (ranging from 0 to 373, median 3), and the total RCR score was 3,104.38 (ranging from 0 to 81.92, median 1.34). Very weak correlations were found: 0.086 (95% CI, 0.038–0.134) between AASs and citations, 0.269 (95% CI, 0.223–0.317) between AASs and RCRs, and 0.188 (95% CI, 0.139–0.234) between AASs and CNCIs.

### 3.5. The Correlation Analysis by Years

Spearman rank correlation coefficient was calculated between citations and the AASs by years (Table 5). The results showed very weak correlations between AASs and citations, AASs and CNCIs, and AASs and RCRs in all years.

### 3.6. The Correlation between AASs and Impact Factors

The correlations between AASs and impact factors were individually analyzed based on years. Weak correlations were found in most of the years (Table 6). Spearman rank correlation coefficient was calculated between the impact factors and AASs based on journals (Table 7). Weak correlations were found for the Australian Critical Care, International Journal of Mental Health Nursing, International Journal of Nursing Studies, Journal of Advanced Nursing, and Nurse Education Today.

## 4. Discussion

In the current study, the Altmetric and traditional bibliometric of journal articles published in 20 top nursing journals were analyzed. A very weak correlation between AASs and citations was found. AAS was also found to be correlated with RCR and CNCI, which were novel metrics of research influence based on citation times. The results suggest that traditional bibliometrics and AAS cannot be used interchangeably but rather complementarily when assessing the impact of journal articles in the nursing field.

Further analysis by journals and publication years found that the International Journal of Nursing Studies (IJNS) had more correlation between the AASs and citations, and no correlation was found in the Nursing Ethics. This difference might be due to the difference in the breadth of journal topics since one is a comprehensive nursing journal and the other is a subspecificity nursing journal. Articles from IJNS with high online attention might also impact the scientific community and thus increase citations. However, articles from Nursing Ethics, which are likely to be controversial articles for the public to garner public interest, thereby increasing AAS, while they might not impact the scientific community in the same manner [12].

With the COVID-19 pandemic [22], more training programs in the nursing practice field have been performed via the social media platforms and other online platforms. That should be of interest to nursing managers, nurses, and journals. It indicates that the social media and other online platforms would not only help the scientific community but also help the public community, especially during the COVID-19 pandemic period [23]. Implementing new research works on the online platforms will contribute to these results and further help the scientific community.

The study has several strengths. First, the current study was a study with large sample size analyzing alternative metrics and bibliometrics based on articles in top nursing journals. Second, it analyzed the AASs with 3 citation-based metrics and found weak correlations between these parameters. Third, we analyzed the correlation by journals, which was a unique contribution to the nursing field. The paper also has several limitations. First, to analyze the precision correlations, we excluded journal articles that missed any data of the four variables (AASs, citations, RCRs, and CNCIs). Second, the correlations might be different when analyzing at a different time. Third, the study only analyzed the journal articles from top nursing journals, and nursing-associated journal articles published in other journals could have different kinds of correlations, especially for those articles published in low-impact factor journals. Fourth, the study did not include other bibliometrics [24, 25], such as the Eigenfactor score and CiteScore; in the future, a new study should be performed to assess these correlations.

In conclusion, the study of articles published in high-impact nursing journals between 2010 to 2019, finds very weak correlations between AAS and citation-based metrics, which suggests promoting journal articles on online platforms may help research, journals, and nurses. Future studies are needed to assess the long-term correlations among these metrics for nursing journal articles.

## 5. Implication to Nursing Management

Understanding the correlation of online impact with traditional bibliometrics of research is critical to nursing practitioners, which in turn helps manage nursing journal articles and research. Nursing managers should develop targeted strategies to increase the online impact of research or nursing practice and increase research impact.

## Figures and Tables

**Table 1:** The impact factors of the included 20 nursing journals from 2010 to 2019.

Journal	2010	2011	2012	2013	2014	2015	2016	2017	2018	2019
Asian Nursing Research	0.133	0.071	0.44	0.418	1.000	0.849	0.768	0.918	1.256	0.988
Australian Critical Care	—	0.973	0.953	1.265	1.562	1.479	1.907	1.930	2.515	2.214
Birth-Issues in Perinatal Care	1.821	2.182	2.926	2.048	1.264	1.867	2.518	2.329	2.129	2.705
BMC Nursing	—	—	—	—	—	—	—	—	—	1.846
European Journal of Cardiovascular Nursing	1.348	1.711	2.042	1.828	1.876	2.491	2.763	2.651	2.497	2.296
Intensive and Critical Care Nursing	—	—	—	—	—	1.214	1.326	1.653	1.652	1.886
International Journal of Mental Health Nursing	1.427	1.071	1.287	2.009	1.950	1.943	1.869	2.033	2.433	2.383
International Journal of Nursing Studies	2.103	2.178	2.075	2.248	2.901	3.561	3.755	3.656	3.570	3.783
International Nursing Review	0.588	1.038	0.939	0.736	0.948	1.073	1.517	1.496	1.562	2.034
Journal of Advanced Nursing	1.540	1.477	1.527	1.685	1.741	1.917	1.998	2.267	2.376	2.561
Journal of Clinical Nursing	1.228	1.118	1.316	1.233	1.255	1.384	1.214	1.635	1.757	1.972
Journal of Nursing Management	1.452	1.181	1.454	1.142	1.500	1.721	1.905	1.912	2.386	2.243
Journal of Nursing Scholarship	1.392	1.490	1.612	1.772	1.636	2.128	2.396	2.662	2.540	2.655
Nurse Education Today	1.113	1.241	1.218	1.456	1.364	1.591	2.533	2.067	2.442	2.490
Nursing Ethics	1.085	0.815	1.210	1.093	1.247	1.469	1.755	1.876	1.957	2.597
Nursing Outlook	1.653	1.522	2.359	1.831	1.588	2.287	2.236	2.425	2.540	2.833
Research in Nursing & Health	1.736	1.708	2.181	1.163	1.267	1.638	1.693	1.762	1.678	2.163
Seminars in Oncology Nursing	—	—	—	—	—	—	—	1.667	1.412	1.330
Women and Birth	—	—	—	1.696	1.573	1.525	2.138	1.822	2.079	2.308
Worldviews on Evidence-Based Nursing	1.429	1.239	1.349	2.318	2.381	1.762	2.103	2.143	2.500	1.991

—: not available.

**Table 2:** The citations, RCRs, CNCIs, and AASs of the included 20 nursing journals [median (range)].

Journal	Citations	RCRs	Category normalized citation impact (CNCI)s	AASs
Asian Nursing Research	9 (0–677)	0.88 (0.08–34.29)	0.9267 (0–26.0303)	1 (0–151)
Australian Critical Care	8 (0–73)	0.83 (0–10.92)	0.6222 (0–8.4983)	3 (0–137)
Birth-Issues in Perinatal Care	12 (0–202)	1.23 (0–11.48)	1.0873 (0–10.717)	4 (0–773)
BMC Nursing	7 (0–78)	1.155 (0–11.67)	0.9076 (0–11.9965)	2 (0–215)
European Journal of Cardiovascular Nursing	11 (0–676)	0.93 (0–36.46)	0.7728 (0–36.5083)	1 (0–206)
Intensive and Critical Care Nursing	8 (0–66)	1.07 (0–7.69)	1.0534 (0–8.3769)	2 (0–76)
International Journal of Mental Health Nursing	10 (0–172)	1.09 (0–13.22)	0.82735 (0–11.0035)	7 (0–220)
International Journal of Nursing Studies	19 (0–582)	1.79 (0–35.94)	1.6983 (0–27.4897)	3 (0–703)
International Nursing Review	9 (0–253)	0.94 (0–23.61)	0.89685 (0–20.285)	2 (0–73)
Journal of Advanced Nursing	12 (0–415)	1.15 (0–25.58)	1.1492 (0–18.9222)	7 (0–1462)
Journal of Clinical Nursing	10 (0–262)	0.98 (0–21.32)	0.9812 (0–20.571)	2 (0–251)
Journal of Nursing Management	11 (0–203)	1.43 (0–17.58)	0.9246 (0–11.235)	1 (0–228)
Journal of Nursing Scholarship	12 (0–301)	1.19 (0–17.95)	1.3087 (0–23.6331)	2 (0–170)
Nurse Education Today	11 (0–770)	1.34 (0–81.92)	1.08825 (0–100.5749)	3 (0–373)
Nursing Ethics	9 (0–132)	1.15 (0–15.36)	1.1756 (0–16.0068)	1 (0–165)
Nursing Outlook	10 (0–184	1.045 (0–15.45)	1.0906 (0–13.1287)	2 (0–670)
Research in Nursing & Health	11 (0–363)	0.915 (0–40.90)	1.1363 (0–22.0488)	1 (0–244)
Seminars in Oncology Nursing	6 (0–230)	0.51 (0–20.55)	0.4514 (0–17.3025)	1 (0–56)
Women and Birth	9 (0–234)	1.07 (0–17.53)	0.8593 (0–8.8129)	3 (0–231)
Worldviews on Evidence-Based Nursing	11 (0–234)	1.18 (0–21.91)	1.0154 (0–20.5276)	2 (0–245)

**Table 3:** Attention received by papers from high-impact journals from 2010 to 2019 stratified by different resources.

Year	News mentions	Blog mentions	Policy mentions	Patent mentions	Twitter mentions	Peer review mentions	Weibo mentions	Facebook mentions	Wikipedia mentions	Google + mentions	LinkedIn mentions	Reddit mentions	Pinterest mentions	F1000 mentions	Q&A mentions	Video mentions	Syllabi mentions	Number of mendeley readers	Number of dimensions citations
2010	132	37	252	4	996	5	0	121	26	6	0	0	0	2	0	7	0	84885	36251
2011	153	48	277	26	1406	1	0	115	38	5	0	0	0	1	0	7	0	94199	35584
2012	137	40	286	21	3198	2	0	269	25	20	2	4	0	1	0	9	0	117410	39041
2013	198	46	351	6	5616	5	14	287	39	224	0	1	0	2	0	7	0	137176	41560
2014	294	61	298	1	8873	9	0	363	37	35	0	3	0	1	0	12	0	152609	42582
2015	268	76	246	4	13794	3	0	847	43	32	0	2	0	1	0	14	0	176717	45858
2016	873	85	248	4	29956	7	0	997	60	42	0	2	0	0	0	29	0	196983	43404
2017	660	63	258	0	31567	3	0	1010	29	123	0	3	0	1	1	5	0	189607	39207
2018	940	107	175	5	52475	5	0	952	54	73	0	5	0	3	0	28	0	206479	39251
2019	838	88	128	0	39928	139	0	517	33	9	0	7	0	4	0	13	0	162787	26578

**Table 4:** The correlations between citation and AAS, CNCI and AAS, RCR, and AAS based on journals.

Journal	Citation and AAS		CNCI and AAS		RCR and AAS	
	Rho	*P* value	Rho	*P* value	Rho	*P* value
Asian Nursing Research	0.284	0.002	0.240	0.011	0.234	0.014
Australian Critical Care	0.115	0.043	0.309	<0.001	0.342	<0.001
Birth-Issues in Perinatal Care	0.234	<0.001	0.311	<0.001	0.347	<0.001
BMC Nursing	0.286	<0.001	0.244	0.003	0.204	0.014
European Journal of Cardiovascular Nursing	0.107	0.023	0.225	<0.001	0.233	<0.001
Intensive and Critical Care Nursing	0.143	0.005	0.239	<0.001	0.234	<0.001
International Journal of Mental Health Nursing	0.012	0.756	0.233	<0.001	0.315	<0.001
International Journal of Nursing Studies	0.207	<0.001	0.267	<0.001	0.371	<0.001
International Nursing Review	0.207	<0.001	0.226	<0.001	0.268	<0.001
Journal of Advanced Nursing	−0.022	0.299	0.105	<0.001	0.209	<0.001
Journal of Clinical Nursing	0.133	<0.001	0.230	<0.001	0.312	<0.001
Journal of Nursing Management	0.158	<0.001	0.206	<0.001	0.239	<0.001
Journal of Nursing Scholarship	0.164	<0.001	0.246	<0.001	0.209	<0.001
Nurse Education Today	0.086	0.001	0.188	<0.001	0.269	<0.001
Nursing Ethics	0.025	0.600	0.055	0.247	0.008	0.860
Nursing Outlook	0.255	<0.001	0.327	<0.001	0.316	<0.001
Research in Nursing & Health	0.152	0.006	0.174	0.002	0.200	<0.001
Seminars in Oncology Nursing	0.246	0.001	0.238	0.001	0.295	<0.001
Women and Birth	0.111	0.005	0.248	<0.001	0.275	<0.001
Worldviews on Evidence-Based Nursing	0.245	<0.001	0.265	<0.001	0.295	<0.001

**Table 5:** Spearman rank correlation coefficient between citation, CNCI, RCR and AAS from 2010 to 2019.

Year	Citation and AAS		CNCI and AAS		RCR and AAS	
	Rho	*P* value	Rho	*P* value	Rho	*P* value
2010	0.293	<0.001	0.262	<0.001	0.275	<0.001
2011	0.327	<0.001	0.318	<0.001	0.306	<0.001
2012	0.238	<0.001	0.231	<0.001	0.219	<0.001
2013	0.278	<0.001	0.258	<0.001	0.262	<0.001
2014	0.301	<0.001	0.284	<0.001	0.277	<0.001
2015	0.285	<0.001	0.253	<0.001	0.271	<0.001
2016	0.259	<0.001	0.207	<0.001	0.241	<0.001
2017	0.280	<0.001	0.236	<0.001	0.268	<0.001
2018	0.275	<0.001	0.240	<0.001	0.269	<0.001
2019	0.242	<0.001	0.208	<0.001	0.246	<0.001

**Table 6:** The correlation between impact factors and AASs from 2010 to 2019.

Year	Rho	*P* value
2010	0.156	<0.001
2011	0.075	0.025
2012	0.125	<0.001
2013	0.186	<0.001
2014	0.165	<0.001
2015	0.190	<0.001
2016	0.078	0.001
2017	0.203	<0.001
2018	0.183	<0.001
2019	0.141	<0.001

**Table 7:** The correlation between impact factors and AASs based on journals.

Journal	Rho	*P* value
Asian Nursing Research	−0.086	0.371
Australian Critical Care	0.550	<0.001
Birth-Issues in Perinatal Care	−0.050	0.312
BMC Nursing	—	—
European Journal of Cardiovascular Nursing	0.208	<0.001
Intensive and Critical Care Nursing	0.166	0.003
International Journal of Mental Health Nursing	0.590	<0.001
International Journal of Nursing Studies	0.307	<0.001
International Nursing Review	0.121	0.023
Journal of Advanced Nursing	0.526	<0.001
Journal of Clinical Nursing	0.287	<0.001
Journal of Nursing Management	0.142	<0.001
Journal of Nursing Scholarship	0.153	0.001
Nurse Education Today	0.352	<0.001
Nursing Ethics	0.110	0.021
Nursing Outlook	0.168	<0.001
Research in Nursing & Health	−0.058	0.299
Seminars in Oncology Nursing	−0.144	0.108
Women and Birth	0.214	<0.001
Worldviews on Evidence-Based Nursing	0.060	0.265

—: not available.

## Data Availability

The data used to support the findings of this study are available from the corresponding author upon request.
